# Swimming exercise to control precocious maturation in male seabass (*Dicentrarchus labrax*)

**DOI:** 10.1186/s12861-018-0170-8

**Published:** 2018-04-12

**Authors:** Marco Graziano, Raul Benito, Josep V. Planas, Arjan P. Palstra

**Affiliations:** 1 Department of Physiology and Immunology, School of Biology, University of Barcelona, Diagonal 643, 08028 Barcelona, Spain; 2 Wageningen Marine Research, Wageningen University & Research, Korringaweg 5, 4401 NT Yerseke, The Netherlands; 3 Wageningen Livestock Research, Wageningen University & Research Animal Breeding and Genomics, PO Box 338, 6700 AH Wageningen, The Netherlands

**Keywords:** Aquaculture, Fish endocrinology, Precocious maturation, Puberty, Optimal swimming speed

## Abstract

**Background:**

Male European seabass, already predominant (~ 70%) in cultured stocks, show a high incidence (20–30%) of precocious sexual maturation under current aquaculture practices, leading to important economic losses for the industry. In view of the known modulation of reproductive development by swimming exercise in other teleost species, we aimed at investigating the effects of sustained swimming on reproductive development in seabass males during the first year of life in order to determine if swimming could potentially reduce precocious sexual maturation.

**Methods:**

Pre-pubertal seabass (3.91 ± 0.22 g of body weight (BW)) were subjected to a 10 week swimming regime at their optimal swimming speed (U_opt_) in an oval-shaped Brett-type flume or kept at rest during this period. Using Blazka-type swim tunnels, U_opt_ was determined three times during the course of the experiment: 0.66 m s^− 1^ at 19 ± 1 g BW, 10.2 ± 0.2 cm of standard length (SL) (week 1); 0.69 m s^− 1^ at 38 ± 3 g BW, 12.7 ± 0.3 cm SL (week 5), and also 0.69 m s^− 1^ at 77 ± 7 g BW, 15.7 ± 0.5 cm SL (week 9). Every 2 weeks, size and gonadal weight were monitored in the exercised (*N* = 15) and non-exercised fish (*N* = 15). After 10 weeks, exercised and non-exercised males were sampled to determine plasma 11-ketotestosterone levels, testicular mRNA expression levels of genes involved in steroidogenesis and gametogenesis by qPCR, as well as the relative abundance of germ cells representing the different spermatogenic stages by histological examination.

**Results:**

Our results indicate that sustained swimming exercise at U_opt_ delays testicular development in male European seabass as evidenced by decreased gonado-somatic index, slower progression of testicular development and by reduced mRNA expression levels of follicle stimulating hormone receptor *(fshR)*, 3-beta-hydroxysteroid dehydrogenase (*3βhsd)*, 11-beta hydroxysteroid dehydrogenase *(11βhsd),* estrogen receptor-beta *(erβ2)*, anti-mullerian hormone *(amh),* structural maintenance of chromosomes protein 1B *(smc1β)*, inhibin beta A (*inhba)* and gonado-somal derived factor 1 *(gsdf1)* in exercised males as compared with the non-exercised males.

**Conclusions:**

Swimming exercise may represent a natural and non-invasive tool to reduce the incidence of sexually precocious males in seabass aquaculture.

## Background

A common feature shared by all juvenile vertebrates is the quiescent state of activity of the brain-pituitary-gonad (BPG) axis that characterizes the post-embryonic developmental period until the onset of puberty. Puberty is then characterised by the first spermatogenic event in males and the start of vitellogenesis in females [1]. In teleosts, the general scheme that drives puberty (GnRH/LH-FSH/sex steroids) is well understood but the factors that trigger the shift from an inactive to a competent BPG axis are not yet fully explained [2–5]. Metabolic signals and environmental stimuli are known to play a fundamental role in triggering puberty [3, 6]. The identification of the exact factors that regulate the onset of puberty in teleosts could have major implications for the aquaculture industry. Early gonadal maturation is a relevant problem in several commercially important species such as Atlantic salmon (*Salmo salar*) [7], sea bream (*Sparus aurata*) [8], flatfishes [9], Tambaqui [10] and European seabass (*Dicentrarchus labrax*) [11, 12]. In seabass aquaculture, the high incidence (20 to 30%) of precocious sexual maturation in males during the first year of life places a critical restraint to production parameters. In this gonochoristic species, wild males normally reach puberty after the second year of life [2, 13]. Precocious maturation of males, already predominant in the fish stock (up to 70%), results in non-marketable fish due to the fact that growth and feed utilization are negatively affected by the energy invested in the production of gametes. Moreover, seabass precocious males have shown to exhibit lower sperm quality and viable milt production as compared to normal-maturing ones [14], thus affecting the fertilization success. Consequently, they reduce the availability of offspring from a given broodstock and create an impact on breeding programs and management in both research-related and commercial aquaculture.

In the last 10 years, efforts to modulate the onset of puberty in seabass have focused on manipulating the photoperiod. Seabass males subjected to manipulated photoperiod for 12 months after hatching show a decreased incidence of precocious maturation [13] resulting in a major investment in somatic growth [15]. In addition, it has recently been reported that in this species there is a definite temporal window (200 days after hatching) when the pineal gland is responsive to photoperiod manipulation and gametogenesis takes place [16]. Although photoperiod manipulation is effective, it may be not efficient in terms of commercial applications (e.g. high economic expenditure, difficult to apply in semi extensive and extensive aquaculture systems). Thus, other methods to control the onset of puberty in this species may be more applicable.

In other species, such as the European eel (*Anguilla anguilla*) and the rainbow trout (*Oncorhynchus mykiss*), swimming affects the beginning of puberty [17, 18]. At the end of its life, the European eel embarks on a 6000-km reproductive homing migration from the European coasts to the spawning ground in the Sargasso Sea. In autumn, the eels swim downstream the rivers and then enter the sea while they may be old but still in a pre-puberistic state [9]. Eel represents a complex model for sexual maturation as swimming exercise in seawater suppresses hepatic vitellogenesis [18] while spermatogenesis is stimulated [19]. Also in migratory rainbow trout, swimming exercise suppressed vitellogenesis [17]. As shown by these examples, a reproductive delay, as induced by exercise, may extend the period of growth and prevent the occurrence of precocious sexual maturation. Swimming exercise may thus represent a way to significantly control puberty in farmed fish [20].

However, no information is available on the possible modulatory effects of swimming on sexual maturation in seabass. Therefore, in this study we have investigated the effect of long-term swimming exercise on gonadal development in male European seabass.

## Methods

### Fish and conditions

The experiments were performed at the fish facility of Wageningen Marine Research, formerly IMARES (Yerseke, The Netherlands). Juvenile seabass (*Dicentrarchus labrax)* (*N* = 1000; 3.91 ± 0.22 g of body weight (BW) [mean ± SEM]; range 2.09–6.68 g) were purchased from a commercial hatchery (Ecloserie Marine de Gravelines, Gravelines, France). Upon arrival at the facilities, fish were transferred to three 800 L circular tanks, connected as a single Recirculating Aquaculture System (RAS) supplied with filtered natural seawater from the Oosterschelde estuary (salinity: 30.1 ‰; pH: 7.7) that was heated to a temperature of 25 °C (water renewal rate: 20–30% per week). Fish were kept under 18 L/6D photoperiod and were fed three times per day on commercial 1.5 mm dry pellets (Skretting Perla MP: 50% crude protein, 15% crude fat, 2.3% fiber, 10% ash, 2% calcium, 0.7% sodium, 1.5% phosphor) ad libitum. Light intensity at the water surface was 40–50 lx. Experiments were started after 1 month of acclimatisation.

### Swimming training in the swim flume

Juvenile seabass were subjected to a 10-week swimming training experiment in a 3600 L oval-shaped Brett-type flume (3.0 × 2.0 × 1.0 m) [21], located in a 100 m^2^ air-conditioned room kept at 20 °C. The flume was operated as RAS like the three 800 L tanks and every hour the entire water volume of the flume was pumped over a drum filter (model HDF 501-1P, Hydrotech AB, Vellinge, Sweden), subsequently through a trickle filter (model Fleuren and Nooijen BV, Nederweert, the Netherlands), through a 200 L biological moving bed biofilm reactor (MBBR), through a protein skimmer (model Sander Aquarientechnik, Uetze-Eltze, Germany) and finally into a 400 L sump before being pumped again into the flume. Water from the sump was continuously pumped via a bypass over a UV-filter (type Proclear UV30 Advantage, Tropical Marine CenterLtd. Hertfordshire, UK) and a heat exchanger (Maxicool XGL18HDA, Maxicool BV, Wessem, the Netherlands, modified by Climate4u.nl, Valkenswaard, The Netherlands) in order to reduce bacterial load and maintain a stable temperature profile, respectively. The flume was described in detail by Palstra et al. [22]. In short, on one of the straight ends, a compartment (200 × 70 cm) was created by two mesh fences of green polyester coated steel (11 mm mesh size) that was then divided in two parallel sub compartments by a PVC sheet (10 mm thick), thus each measuring 200 cm in length (x), 35 cm in width (y) and 70 cm in depth (z) with a total volume of 525 L. The inner sub-compartment housed the resting fish (*N* = 100) and a water flow was produced by a pump (Aqua Ocean Runner OR 6500, Aqua Medic, Loveland, CO, USA) just in order to secure good water quality. The outer sub-compartment housed the swimming fish (N = 100) where a basic water flow was generated by a Speck pump (Badu 90/13, 0.55 kW with a 13 m^3^ s^− 1^ capacity; Speck Pumps, Jacksonville, USA). The additional water flow to force fish to swim at their optimal swimming speed was generated by an impeller connected to an electric motor (model Kleedrive MS2 132 M-4 B3 (7.5 w), Brd. Klee A/S, Albertslund, Denmark). The motor was powered by an industrial inverter (IP66, model n^0^ BFI-E2–34-0180-3F4#, Bejer Electronics, Malmo, Sweden) with an adjustable alternating current (AC) from 0 to 50 Hz. Inverter frequencies from 2.5 to 8.5 Hz were linked to flow speeds that were measured using a Vectrino acoustic Doppler velocimeter (ADV; NortekAS, Rud, Norway) in three dimensions (velocities *u, v* and *w* in directions *x, y* and *z* respectively) for 10 s with a sampling rate of 10 Hz. Horizontal water velocity (*u*) in the swim compartment increased proportionally to the AC-frequency of the inverter. Water parameters in the flume were kept the same as in the 800 L stocking tanks during the whole 10-week experiment.

### Determination of the optimal swimming speed in swim tunnels

Fish were induced to swim at their optimal swimming speed (U_opt_). Over the course of the experiment U_opt_ was determined three times to adjust the applied flow velocity in the flume: at weeks 1, 5 and 9. For this purpose, two 127 L Blazka-type swim tunnels were used (described in detail by Van den Thillart et al. [23]). The day before the swim tunnel experiment, one seabass was introduced in each of the tunnels for overnight acclimatisation at the same light regime and water conditions as described before. Water velocity overnight was kept at 0.1 m s^− 1^ to ensure sufficient water quality. The following morning, a critical swimming speed (U_crit_) test was executed by starting at a swimming speed of 0.1 m s^− 1^ and then increasing the swimming speed by 0.1 m s^− 1^ every 10 min until the maximum swimming speed of 1 m s^− 1^ was reached. All experimental fish were able to swim the full range of speeds and the U_crit_ test was finished after swimming 10 min at 1 m s^− 1^, so U_crit_ values were all > 1 m s^− 1^. After the U_crit_ test, fish were left recovering at the minimum speed (0.1 m s^− 1^) for 120 min. Subsequently, an U_opt_ test was performed and oxygen (O_2_) content was measured using for each tunnel a bypass with a galvanic oxygen electrode, both applied in a 4-channel respirometry system (DAQ-PAC-G4; Loligo Systems Aps, Tjele, Denmark; Palstra et al., [22]). The decrease in O_2_ content which equals O_2_ consumption was measured at 4 different swimming speeds: 0.25, 0.50, 0.75 and 1 m s^− 1^ in random order. Fish swam for 45 min at each speed after which they recovered at minimal speed for 15 min. The tunnels recirculated water during the 45 min swimming periods to allow O_2_ measurements after which they were flushed during recovery. O_2_ data from minutes 15 to 45 at each speed were used for analyses. This protocol was repeated until O_2_ was measured for all four swimming speeds. Background O_2_ consumption with only water and no fish inside the tunnels was determined for data correction when found significant.

The percentual decline of oxygen content was used to directly calculate the cost of transport (COT) according to the formula:

1 $$ COT=\frac{{\Delta  sat}_{(t)}\bullet {mg}_{O_2}}{m\bullet \Delta  d} $$

where: ∆*sat*_(*t*)_ is the % decline in oxygen saturation during the measurement interval, ${mg}_{O_2}$ is the amount of oxygen in mg per % saturation under the given conditions, *m* is the body mass of the fish in kg and ∆*d* is the covered distance in m. COT data at 100% U_crit_ were not considered when plotting the polynomial trend line due to the considerable anaerobic contribution to swimming at critical speeds. By equaling the first derivative of the polynomial function that described the relation between COT and the swimming speed *U* (0.25, 0.50 and 0.75 m s^− 1^) to zero, the *U*_opt_ was calculated (also Palstra et al. [24]). The solid blocking effect (SBE; [25]) was negligible. After each series of swim tunnel experiments, the newly determined U_opt_ was adopted in the swim flume.

After each swim tunnel experiment, experimental fish were anesthetized (Clove oil, diluted 1:10 in absolute ethanol and then used at 2 ml l^− 1^ water) and 1 ml of blood was extracted from the caudal vein using heparin-flushed (10.000 IU ml^− 1^) 1 ml syringes that were immediately placed on ice after use. Hematocrit (Hct) values were measured in 9 μl whole blood samples as triplicates per fish using a microcentrifuge (Spincrit microhematocrit centrifuge, Indianapolis, US). Fish were then euthanized by a blow on the head and measured for standard length (SL) and BW.

### Sample collection

A subsample of fish (*N* = 15) was sampled at the start of the swim-experiment and subsamples of the exercised fish (*N* = 15) and the non-exercised controls (*N* = 15) were sampled every 2 weeks. At each sampling time, fish were anaesthetised, euthanized by decapitation and measured for SL and BW. Gonads were dissected and weighed to the nearest 0.01 g as gonad weight (GW). The gonadosomatic index (GSI) was then calculated as (GW BW^− 1^)*100%. Only at week 10, 1 ml of blood was extracted as described above, centrifuged at 4 °C (5 min at 9.500 x g) and plasma was stored at − 80 °C for subsequent analysis of steroid levels. One of the two gonads from fish in week 10 was stored in RNAlater (Ambion) at − 20 °C. The remaining gonad was stored in formalin (4% buffered formaldehyde) and kept at 4 °C for histological analyses. All samples were sent to the University of Barcelona for analyses.

### Plasma 11- ketotestosterone

To quantify the levels of 11-ketotestosterone (11-KT) in plasma samples from males sampled in week 10, a commercial kit (11-keto Testosterone EIA Kit, Cayman Chemical Company, USA) was used following the manufacturer’s specifications.

### Gonad histology

The gonad samples in formalin were washed for 24 h in PBS, dehydrated in an ascending series of ethanol concentrations and then cleared in xylene and embedded in paraffin (pfm medical ag, Köln, Germany) for 1 h at 50 °C. Subsequently, 6 μm thick sections were cut using a microtome (model Leica 2235, Leica biosystems inc, Ontario, Canada), were mounted on polylysine slides, dried overnight at 37 °C, and stored at 4 °C. Sections were stained with hematoxylin-eosin, according to standard histological procedures [26, 27], and mounted using DPX medium (Scharlab, Barcelona, Spain). The sections were analysed under a bright field microscope (Olympus CX23, Olympus Deutschland GmbH, Hamburg, Germany) to determine the sex and maturity stage of the gonads. Pictures of the histological sections were taken using a 12.5 Px DP70 digital camera (Olympus Deutschland GmbH, Hamburg, Germany). Phenotypic sex of these sections could be determined by microscopic examination [28]. Testicular samples were selected for further analyses. Stages of spermatogenesis were classified according to Begtashi et al. [11]. Germ cells were classified according to their topography, morphology, size and nuclear characteristics. Subsequently, the testis area covered by a particular cell type was calculated as percentage of the whole gonadal area in six individual males per group using the ImageJ Plugin Analyze (ImageJ software (Rasband, W.S., ImageJ, U.S. National Institutes of Health, Bethesda, Maryland, USA, http://imagej.nih.gov/ij/, 1997–2016). Gonadal area fractions were estimated by placing a 48 point grid on the image (microphotography) and then characteristic cell types were marked and counted trough the “cell counter” ImageJ tool according to Butts et al. [29]. The lumen’s portion and the area that was not covering any tissue were excluded from the analysis.

### cDNA synthesis and quantitative Real Time PCR

Testicular samples of exercised (*N* = 10) or non-exercised (*N* = 8) juvenile seabass at week 10, preserved in RNAlater, were processed for RNA purification, and cDNA synthesis and quantitative real time PCR (qPCR) were performed as previously described [30–32]. Briefly, total RNA was extracted with TRI Reagent Solution (Applied Biosystems) following the manufacturer’s instructions and treated with DNaseI (amplification grade, 1 unit/ μg RNA; Invitrogen). SuperScript III RNase H− Reverse Transcriptase (Invitrogen) was used to synthesise first strand cDNAs with oligo (dT)15 Primer (Promega) from 1 μg of total RNA at 50 °C for 60 min. Candidate genes involved in testicular development and function (Table 1) were selected from the GenBank nucleotide database and from the expressed sequence tags (ESTs) deposited in the Aquasea database (Aquagenomics Consortium; available to J. Planas).

**Table 1:** Primers

Gene	FW/RV	Sequence (5′ > 3′)	Length	Tm	GC%	Self complementarity	Self 3′ complementarity	Product length	AC	Authors
*igf1*	Forward primer	GGCTTGGCTAATCTAACTGGCTTC	24	61.75	50.00	4.00	0.00	160	GQ924783.1	Crespo et al. 2013
	Reverse primer	TCGCTCAGGGGTTTCATCACTAC	23	62.24	52.17	3.00	0.00			
*amh*	Forward primer	TGCAGAGCAAAGCCTGAAAG	20	59.04	50.00	4.00	1.00	126	AM232701.1	Diaz *et* Piferrer, 2015
	Reverse primer	TCAACGGGGAACAAAGACAA	20	57.58	45.00	2.00	0.00			
*fshr*	Forward primer	ACTCCACCTCCATCATCTGC	20	59.16	55.00	3.00	2.00	177	AY642113.1	Rocha et al. 2007
	Reverse primer	AACGGGGAACAGTCAGTTTG	20	58.32	50.00	5.00	1.00			
*bmp15*	Forward primer	GGCAGATTTGATGGGTCATT	20	56.05	45.00	4.00	1.00	117	AM933668.1	Halm et al. 2008
	Reverse primer	CTTTAACAGGAACGGCGAAG	20	57.12	50.00	4.00	0.00			
*erβ2*	Forward primer	GAGCTGGAGAGCAGGAACAA	20	60	55.00	4.00	0.00	61	AJ489524.1	Designed, partial sequence from Halms et al. 2007
	Reverse primer	AGACCAGAGCATCGATCACC	20	60	55.00	6.00	2.00			
*gsdf1*	Forward primer	ACAGAGCTGCCTTGCAATCC	20	60.96	55.00	6.00	4.00	99	JQ755271.1	Crespo et al. 2013
	Reverse primer	TCTTGTATGACAAAGCCTGCC	21	58.56	47.62	6.00	1.00			
*arα*	Forward primer	CCCCGGATCTTGTGTTCA	18	60	55.56	4.00	2.00	70	AY647256.1	Designed, comlete sequence from Blazquez *et* Piferrer, 2015
	Reverse primer	TTCATCCGTATGCAGTGTTCA	21	60	42.86	6.00	2.00			
*3βhsd*	Forward primer	CACCCTACAAGAGCTACGAGGA	22	60	54.55	4.00	0.00	96	JQ861952.1	Designed, partial sequence from Mazon *et* Gomez, 2012
	Reverse primer	CCAGAACCCAGAGGACCAG	19	60	63.16	3.00	1.00			
*11βhsd*	Forward primer	GGAAATGCTGGCAACCAC	18	60	55.56	4.00	2.00	65	AF449173.2	Designed, complete sequence from Mylonas et al. 2007
	Reverse primer	CACGAACACGGAGCAACA	18	60	55.56	2.00	0.00			
*smc1β*	Forward primer	TTCAGACCGATGGACAACCT	20	60	50.00	3.00	0.00	87	KF699104.1	This study
	Reverse primer	GGCGGGTTTGTAACTGTGAA	20	60	50.00	3.00	1.00			
*inhba*	Forward primer	TCATCAAGAAGGACATCCAGAA	22	58.62	40.91	4.00	1.00	62	HE967317.1	Designed, complete sequence from Garcia-Lopez et al. 2012
	Reverse primer	GGTTCGGTGTTTACGAGCAG	20	59.21	55.00	3.00	1.00			
*l17*	Forward primer	CTGGCTTGCCTTTCTTGACT	20	60	50.00	4.00	1.00	201	AF139590	Versamos et al. 2006
	Reverse primer	GAGGACGTGGTGGTTCATCT	21	60	55.00	4.00	0.00			

Forward (FW) and reverse (RV) primer sequences (5′ > 3′), their characteristics (length, guanine-cytosine content (GC%)) and qPCR conditions (melting temperature Tm, self-complementarity, self-complementarity of the 3′ end and product length) for the seabass gene expressions analysed in this study: insuline-like growth factor 1 (igf 1), anti-mullerian hormone (amh), follicle stimulating hormone receptor (fshr), bone morpho-genetic protein (bmp15), estrogen receptor- beta (erβ2), gonado-somal derived factor 1 (gsdf1), androgen receptor-alpha (arα), 3-beta-hydroxysteroid dehydrogenase (3βhsd), 11-beta hydroxysteroid dehydrogenase (11βhsd), structural maintenance of chromosomes protein 1B (smc1β), inhibin beta a (inhba), ribosomal protein L 17 (i17) and the literature reference reporting on the specific primers. Authors and accession numbers (AC) of the used primers are indicated

Genes were selected according to their role in the three main processes of interest: testicular steroidogenesis, sertoli cell’s function / spermatogonial proliferation, and progression of gonadal maturation. The markers involved in testicular steroidogenesis were represented by *3-beta-hydroxysteroid dehydrogenase* (*3βhsd*), *11-beta hydroxysteroid dehydrogenase* (*11βhsd*), indispensable for the production of 11-KT and, by *androgen receptor-alpha* (*arα*) and *estrogen receptor-beta* (*erβ2*). The genes involved in sertoli cell’s function and spermatogonial proliferation that were chosen were *gonado-somal derived factor 1* (*gsdf1*) [33] and *bone morpho-genetic protein* (*bmp15*), proposed as a sperm quality regulator and with expression restricted to germ cells [34]. In the same group are also *follicle stimulating hormone* (*fshr*), the Fsh cognate receptor, the *anti müllerian hormone* (*amh*), and the *insuline-like growth factor 1* (*igf1*) that besides its role as mediator of growth hormone in vertebrates also stimulates DNA synthesis in spermatogonia and is necessary for the continuation of spermatogenesis [35]. Finally, the genes involved in the progression of gonadal maturation that were chosen were *structural maintenance of chromosomes protein 1b* (*smc1β*), a gene required for the maintenance of meiotic cohesion [36], and *inhibin beta a* (*inhba*) a gene that participates in the regulation of Fsh synthesis and cell proliferation and differentiation [37, 38].

In order to isolate partial cDNAs encoding seabass 3-beta-hydroxysteroid dehydrogenase (*3βhsd*), 11-beta-hydroxysteroid dehydrogenase (*11βhsd*), anti müllerian hormone (*amh*), follicle stimulating hormone (*fshr*), androgen receptor isoform a (*ar*), the gonadosomal derived factor (*gsdf1*), estrogen receptor isoform B (erβ2), structural maintenance of chromosomes protein 1b (*smc1β*), bone morphogenetic protein 15 (*bmp15*) and insulin-like growth factor 1 (*igf1*), oligonucleotide primers were designed. Primer design for the selected candidate was done using the Roche Universal Probe Assay Library except for those for which primer sequence was already published: *fshr* [39], *amh* [40], *gsdf1* [32] and L17 (*l17*) [41] *smc1β* primers were designed using the conserved region of the partial *Solea senegalensis* mRNA sequence available in GenBank. Each qPCR was run in triplicate in 96-well plates using a MyiQ Real Time PCR Detection System (Bio-Rad Laboratories, Inc., Hercules, California, USA). The qPCR amplification reaction mixture, with a final volume of 10 μl, contained 2 μl of cDNA diluted 1:10 to 1:100 depending on the transcript. Specifically, a cDNA dilution of 1:100 was used for *gsdf1* and *smc1β;* for all other qPCR reactions cDNA had a dilution of 1:10. Relative gene expression to the reference gene *l17* was determined using the iQ5 Optical System software 2.1 (Bio-Rad). Ten-fold dilution series, from the most to the least concentrated cDNA samples, were used to test primer efficiencies. Primer efficiencies ranged from 95.6 to 103.0% and excluded the presence of relevant sub-optimal annealing temperatures; poorly designed primers; the formation of amplicons with secondary structures; and occurrence of primer-dimers, that may have affected our results. To determine the precision of the qPCR assay, intra-assay (repeatability) and the inter-assay (reproducibility) fidelity levels, average CT values and SDs were used and reported as coefficients of variation (CVs, SD/mean × 100%). Coefficient of variations (CV%) of triplicates was checked to be smaller than 1. When CV% was found to be higher than 1, duplicate measurements were used if possible. When also the use of duplicates was not appropriate, the entire data point was omitted from the analysis. CV% values were also calculated in all the replicates from the two groups, both intra and inter assays. Reference gene *l17* CV% was 0.78%, for all the other genes analysed CV% values ranged from 0.85 to 3.12%. Inter assays variability showed a CV% value of 2.19%. Primer sequences, characteristics and qPCR conditions and product length for the seabass genes analyzed in this study are shown in Table 1.

### Statistical analyses

After collecting the data, statistical analysis was performed to search for differences between the dependent factors of individuals from both groups (RESTERS and SWIMMERS). All the data obtained, except for gene expression and GSI, were analyzed with the software SPSS Statistics 17.0 (SPSS Inc. Released 2008. SPSS Statistics for Windows, Version 17.0. Chicago, USA). BW, SL and 11-KT values were normally distributed (D’Agostino Pearson omnibus normality test). For 11-KT values, significant differences between the treatments were determined by performing a t-test. For BW and SL, two-way ANOVA was performed according to the model: Week (repeated factor); Treatment (Rest vs Swim); Week x Treatment interaction, and Residual error. The GSI values were not normally distributed and were instead compared among treatments with a Mann Whitney U test followed by a Benjamini-Hochberg procedure for false discovery rate correction to exclude type I errors. Using three replicates per sample, relative gene expression values (∆C_T_) were obtained using the iQ5 Optical System software 2.1 (Bio-Rad Laboratories, Inc., Hercules, California, USA) as indicated in the calculation spreadsheet provided by the manufacturer and derived from the algorithms outlined by Vandesompele et al.*,* [42]. Expression values were then normalized to the control group by dividing the individual gene expression values from both groups by the average expression value of the control group. Statistically significant differences (*P* < 0.05) were determined by performing a Mann-Whitney test using the GraphPad Prism Software for windows version 6.0. (GraphPad Software, La Jolla California USA).

## Results

### Determination of optimal swimming speeds throughout the swimming training experiment

Fish at week 1 weighed 19 ± 1 g, measured 10.2 ± 0.2 cm in SL and had a Hct level of 46 ± 2%. The formula of the COT polynomial trend line was determined as y = 6.9002x^2^ - 9.1459x + 3.67. U_opt_ of fish at week 1 was then calculated at 0.66 m s^− 1^ at a minimal cost of transport (COT_min_) of 0.64 mg kg^− 1^ m^− 1^. Fish at week 5 weighed 38 ± 3 g, measured 12.7 ± 0.3 cm in SL and had a Hct level of 41 ± 1%. The formula of the COT polynomial trend line was determined as 2.9018x^2^ - 4.0186x + 1.7867. U_opt_ of fish at week 5 was then calculated at 0.69 m s^− 1^ at a COT_min_ of 0.40 mg kg^− 1^ m^− 1^. Finally, fish at week 9 weighed 77 ± 7 g, measured 15.7 ± 0.5 cm in SL and had a Hct level of 43 ± 1%. The formula of the COT polynomial trend line was determined as 2.0028x^2^ - 2.7487× + 1.1353. U_opt_ of fish at week 9 was then calculated at 0.69 m s^− 1^ at a COT_min_ of 0.19 mg kg^− 1^ m^− 1^. Thus, although the weight and the length increased and the COT_min_ decreased during the swimming training period, the U_opt_ hardly changed, increasing only from 0.66 to 0.69 m s^− 1^. Therefore, a similar U_opt_ was applied throughout the course of the experiment in the swim flume.

### Effects of swimming training on size and GSI in juvenile seabass

**Fig. 1:**
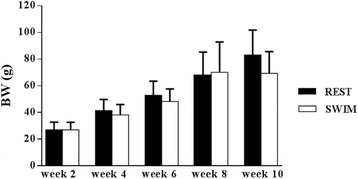
Time-related effects of swimming training on body weight (BW) in juvenile seabass. Resters (REST; *N* = 15) and swimmers (SWIM; *N* = 15) which swam at U_opt_ during a 10-week period in the swim flume. A significant time effect existed (*P* < 0.0001) but no treatment effects

**Fig. 2:**
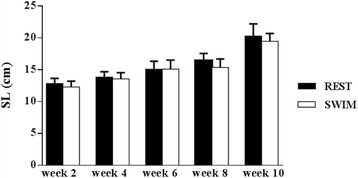
Time-related effects of swimming training on standard length (SL) in juvenile seabass. Resters (REST; *N* = 15) and swimmers (SWIM; *N* = 15) which swam at U_opt_ during a 10-week period in the swim flume. A significant time effect (*P* < 0.0001) and treatment effects existed (*P* = 0.004)

**Fig. 3:**
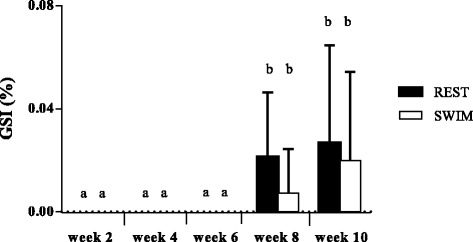
Time-related effects of swimming training on gonadosomatic index (GSI) in juvenile seabass. Resters (REST; *N* = 15) and swimmers (SWIM; *N* = 16) which swam at U_opt_ during a 10-week period in the swim flume. Bars that do not share a letter are statistically different (*P* < 0.05) and represent the mean ± SD of males and females combined

The results of BW, BL and GSI, analyzed for fish at weeks 2, 4, 6, 8 and 10 are shown in Figs. 1, 2 and 3 respectively. BW and SL increased gradually over time throughout the experiment (*P* < 0.0001; Figs. 1 and 2). Only SL showed a significant difference between swimmers and resters (*P* = 0.004) with swimmers being slightly shorter. Gonad weight was lower than 0.01 g for all fish during the first 6 weeks of the experiment and, consequently, GSI values were almost 0 for weeks 2, 4 and 6. GSI increased at weeks 8 and 10 with swimmers showing lower GSI values than resters, although differences between the two groups were not statistically significant (Fig. 3). At week 10, male and female gonads could be discriminated macroscopically and, consequently, the males could be identified to determine the swimming-induced effects on testicular development. The sex ratio at week 10 was 58% males and 42% females. Table 2 provides the results for only the males of the swimming and resting groups at week 10. No statistically significant differences in weight nor in length between the male swimming and resting groups were detected, although male swimmers tended to be smaller than male resters. In addition, no differences in Fulton’s condition factor (K; Fulton, [43]) were observed between male swimmers and resters (Table 2). Importantly, at week 10 the GSI values from the male swimmers were significantly lower than the male resting controls.

**Table 2:** Biometric measurements

	RESTERS	SWIMMERS
N	9	10
BW(g)	83 ± 19	69 ± 16
SL (cm)	16.5 ± 1.11	15.4 ± 1.33
GW (g)	0.038 ± 0.028	0.031 ± 0.021
GSI (%)	0.003 ± 0.003	0.000 ± 0.000*
Hct	48 ± 5	48 ± 5
K	0.018 ± 0.000	0.018 ± 0.000

Body weight (BW), standard length (SL), gonad weight (GW), gonado-somatic index (GSI), haematocrit (Hct), and Fulton’s condition factor (K) values from resting (RESTERS, *N* = 9) and swimming (SWIMMERS, *N* = 10) males at the last dissection (week 10). Values are averages ± SD. * = *P* < 0.05

### Effects of swimming training on 11-KT plasma levels in male juvenile seabass

**Fig. 4:**
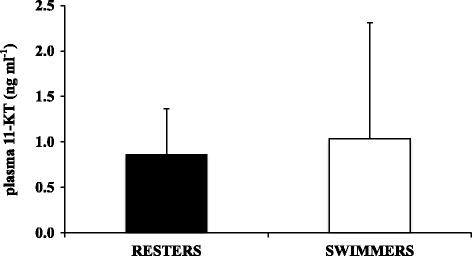
Effects of swimming training on plasma 11-ketotestosterone (11-KT) levels in juvenile male seabass at week 10. Resters (*N* = 9) and swimmers (*N* = 7) which swam at U_opt_ during a 10-week period in the swim flume. Bars represent the mean ± SD

Plasma samples from male seabass sampled at week 10 were used to determine the levels of 11-KT in resting (*N* = 9) and swimming (*N* = 7) males. Our results show that plasma 11-KT levels were similar and not significantly different between swimmers and resters (Fig. 4).

### Effects of swimming training on testicular development in male juvenile seabass

**Fig. 5:**
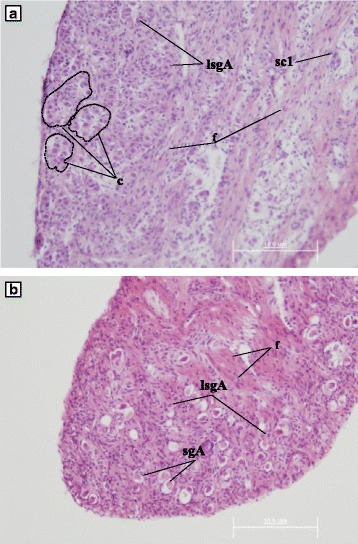
Histological analysis of testicular samples from resters (**a**) and swimmers (**b**). Spermatogonia A (sgA), late spermatogonia A (lsgA), primary spermatocytes (sc1) and fibrocytes (f) are indicated by solid arrows. Spermatic cyst (c) are delineated by dashed lines. Representative images are shown

Stages of spermatogenesis of testes from males sampled at week 10 were determined and classified as stage I-II (immature and early recrudescence, respectively) and stage III (mid recrudescence) according to Begtashi et al. [11]. A high predominance of easily recognizable cysts, late spermatogonia A (lsgA) and a small fraction of primary spermatocytes (sc1) were found in the testes of resting control males as opposed to the testes of swimmers, in which no sc1 and disperse lsgA were identified but that contained a clear predominance of spermatogonia (sgA) (Fig. 5). In the resting males, the percentage of covered gonadal area represented by lsgA was 37% higher than in the swimming males. The characteristic histological condition found in testes from swimmers corresponded to the earlier phases of spermatogenesis (stage I and II) as shown in seabass by Rodriguez et al. [15]. Resting males showed a testicular structure generally consisting in relatively well formed cysts containing SgA, lsgA and disperse sc1 (Fig. 5a). In contrast, the testicular structure of most swimmers consisted of less and smaller cysts, with a predominance of sgA and disperse lsgA (Fig. 5b).

### Effects of swimming training on the expression of genes involved in testicular development and function

**Fig. 6:**
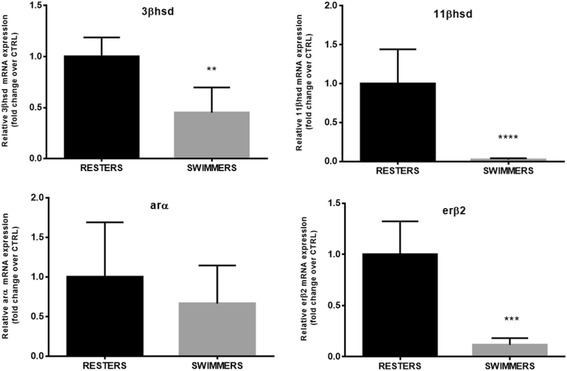
Expression of genes involved in testicular steroidogenesis: *3-beta-hydroxysteroid dehydrogenase* (*3βhsd*), *11-beta hydroxysteroid dehydrogenase* (*11βhsd*), *androgen receptor-alpha* (*arα*) and *estrogen receptor-beta* (*erβ2*) in juvenile seabass males subjected to swimming at U_opt_ (SWIMMERS) or not (RESTERS) for 10 weeks. Data are shown as mean ± SD. Asteriks indicate statistical differences (*P < 0.05; **P < 0.01; ***P < 0.001; ****P < 0.0001)

In view of the observed changes in testicular development in juvenile seabass males subjected to sustained swimming, we proceeded to study the molecular basis of these changes by measuring the mRNA expression levels of genes known to participate in spermatogenesis in seabass. The majority of the analyzed genes involved in testicular development and function showed a significant down-regulation in their mRNA expression levels in testes from swimmers when compared to resters at week 10. Specifically, the mRNA expression of genes involved in testicular steroidogenesis was down-regulated in swimming males when compared to resting males. Expression of *3βhsd*, involved in progesterone synthesis, and *11βhsd*, indispensable for the production of 11-KT, was down regulated in swimming males (*P* = 0.0085 and *P* < 0.0001, respectively) (Fig. 6). Similarly, testicular expression of *erβ2* (*P* = 0.0002) was significantly down-regulated in swimming males as compared to resting ones (Fig. 6). The androgen receptor α (*arα*) was the only one among the steroidogenic functional markers that did not show significant down-regulation in response to swimming, although a trend towards lower mRNA expression values was detected in swimmers (Fig. 6).

**Fig. 7:**
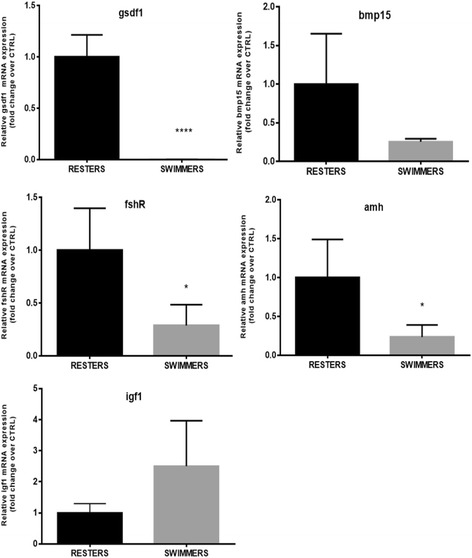
Graphs showing the expression of genes involved in sertoli cells’ function and spermatogonial proliferation: *gonado-somal derived factor 1* (*gsdf1*), *bone morpho-genetic protein* (*bmp15*), follicle stimulating hormone (*fshr*), anti müllerian hormone (*amh*), and *insuline-like growth factor 1* (*igf1*) in juvenile seabass males subjected to swimming at U_opt_ (SWIMMERS) or not (RESTERS) for 10 weeks. Data are shown as mean ± SD. Asteriks indicate statistical differences (*P < 0.05; **P < 0.01; ***P < 0.001; ****P < 0.0001)

The analyzed genes involved in testicular germ cells development and differentiation showed a similar down regulation trend as observed for the steroidogenic markers. The mRNA expression of the Fsh cognate receptor *fshr* and the Anti-Müllerian hormone *amh* was significantly down regulated in swimmers (*P* = 0.0117 and *P* = 0.0205, respectively) (Fig. 7). Furthermore, *gsdf1*, a gene involved in spermatogonial proliferation [33], showed a strong down-regulation (*P* < 0.0001) in swimmers vs. resters (Fig. 7). In swimmers, the mRNA expression levels of *bmp15,* proposed as a sperm quality regulator and whose expression is restricted to germ cells [34], were lower than in resters, even though not significantly (Fig. 7). The only gene that showed up-regulated mRNA expression levels in swimmers over resters, although not significantly, was *igf1*, that beside its role as mediator of growth hormone in vertebrates, stimulates DNA synthesis in spermatogonia and is necessary for the continuation of spermatogenesis [35] (Fig. 7).

**Fig. 8:**
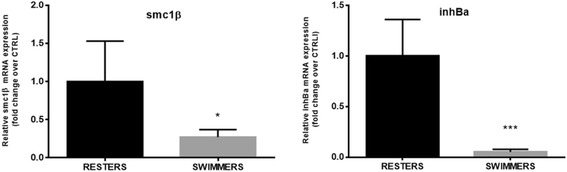
Graphs showing the expression of genes necessary for the progression of gonadal maturation: *structural maintenance of chromosomes protein 1b* (*smc1β*) and *inhibin beta a* (*inhba*) in juvenile seabass males to swimming at U_opt_ (SWIMMERS) or not (RESTERS) for 10 weeks. Data are shown as mean ± SD. Asteriks indicate statistical differences (*P < 0.05; **P < 0.01; ***P < 0.001; ****P < 0.0001)

The expression of genes necessary for the progression of gonadal maturation was down regulated in swimmers. Expression of *smc1β*, a gene required for the maintenance of meiotic cohesion [36], and *inhba*, a gene that participates in the regulation of Fsh synthesis and cell proliferation and differentiation [37, 38], was down-regulated in swimming males as compared to resting males (*P* = 0.0234 and *P* = 0.0002, respectively; Fig. 8).

## Discussion

This is the first study that evaluates the effects of swimming-induced exercise on testicular development in seabass with the aim to reduce the incidence of precocious sexual maturation during farming conditions. Moreover, this study provides insights into the regulatory mechanism of early testicular development on molecular level which is clearly suppressed by swimming-induced exercise, suggesting that forced swimming exercise, during the onset of testicular development, could therefore be used as a natural and non-invasive treatment to inhibit testicular development..

In seabass, early puberty is sensitive to photoperiod manipulation until approximately 200 days after hatching and, consequently, this period has been considered to be the critical time in which the onset of gametogenesis may take place [16]. During this window of time, we hypothesized that swimming-induced exercise may also have a modulatory effect on precocious maturation by inhibiting the gonadotropic production that initiates the gonadal maturation phenomenon. Our results confirm that the selected age and size class of the fish used in this experiment are subject to puberty manipulation as previously observed by other authors [16]. Clearly, further studies are needed to determine whether swimming-induced exercise can have permanent effects on testicular development and whether this approach may represent a potentially cost-effective manipulation of gonadal development in seabass males.

Swimming-induced exercise in our study consisted of 10 weeks of continuous swimming at the optimal swimming speed. Throughout this period, optimal swimming speeds evolved from 0.66 m s^− 1^ to 0.69 m s^− 1^ for juvenile seabass with an initial BW and SL of 19 ± 1 g and 10.2 ± 0.2 cm, respectively, and with a final BW and SL of 77 ± 7 g and 15.7 ± 0.5 cm, respectively, in seawater at 25 °C. These U_opt_ values are higher than the U_opt_ of 0.58 m s^− 1^ as reported by Claireaux et al. [44] for larger seabass (range of 105–248 g BW) swimming in seawater at 26 °C. The effects of swimming-induced exercise are mainly dependent on the applied speed. The stimulating effects of swimming exercise on growth and disease resistance display a clear optimum at a specific speed [45] which is similar to the optimal metabolic swimming speed as determined by respirometry [46]. Therefore we chose U_opt_ as the swimming speed for our study, but it should be considered that speeds lower or higher than U_opt_ will most probably lead to a difference in results. Our results suggest that swimming at optimal swimming speeds for 10 weeks resulted in a suppressive effect on testicular development in this species.

As hypothesized, swimming activity applied during the beginning of spermatogenesis may have inhibited testicular development as a result of increased energy expenditure. The performed histological analysis showed a high predominance of larger and easily recognizable cysts, late spermatogonia A and a small fraction of primary spermatocytes in the non-exercised control fish as opposed to the swimmers in which no primary spermatocytes and late spermatogonia A were detected. This particular histological condition found in swimmers corresponds to an earlier stage of spermatogenesis (I-II) than that found in the non-exercised control fish (III) [16], reminiscent of the delayed testicular development observed in male seabass subjected to long-term exposure to a continuous light photoperiod (Begtashi et al. [11]), and is in accordance with the lower GSI values observed in swimmers. Supporting these findings, the mRNA expression levels of *fshr*, the Fsh cognate receptor, were down-regulated (4-fold) in swimming fish. In the fish testis, *fshr* is predominantly expressed in Sertoli cells and its mRNA expression levels increase in male sea bass during gonadal growth, presumably due to the proliferation of Sertoli cells [47–49]. This receptor has been detected both in the germinal and in the somatic components of the gonads of several fish species such as Japanese eel (*A. japonica*), African catfish (*C. gariepinus*), zebrafish (*D. rerio*), and Senegalese sole (*S. senegalensis*) [50–53]. Fsh is widely considered an essential component in triggering the onset of gonadal maturation in fish [54, 55] and has a crucial role in initiating spermatogenesis by regulating several Sertoli cell functions (e.g., nutritional, structural, paracrine) through its receptor and by inducing the mRNA expression and activity of enzymes involved in the steroidogenic pathway during early spermatogenic stages [48, 53]. Accordingly, the steroidogenic markers *3βhsd* and *11βhsd* selected in this study showed a decreased testicular expression in exercised seabass as compared to the non-exercised controls (50- and 2- fold, respectively), suggesting an impairment of enzymes that are indispensable for the production of gonadal steroids and, consequently, for the entire maturation process [55]. *3βhsd* is specifically involved in the conversion from pregnenolone to progesterone [56] and, a recent study in juvenile male flatfishes, co-localized its presence together with the isoform a of the Fsh receptor in the interstitial component of the testicle, suggesting an Fsh-mediated action that relies on Leydig cell-derived steroids during early testicular development [57]. On the other hand, *11βhsd* is involved in the conversion of testosterone into 11-ketotestosterone (11-KT), a key hormone in the onset of puberty of male teleosts [58, 59], including seabass [47, 60]. Rodríguez et al. determined plasma 11-KT levels during the first sexual maturation in pre-pubertal seabass (i.e. 1-yr old) exposed to expanded (18 months) and compressed (6 months) photoperiods and reported that 11-KT levels were lower under a modified photoperiod than under a natural photoperiod. It has recently been found that, at the onset of puberty in seabass, an increase in 11-KT levels is well correlated with a high incidence of precocious males [61] . However, we did not find any detectable differences in plasma 11-KT levels yet between swimming and resting seabass in this study, in contrast to the observed differences in testicular gene expression and histological data. Similarly, we found no significant differences in androgen receptor *α* (*arα*) mRNA expression levels as a consequence of swimming, thus indicating that androgen action may not be altered by swimming at this developmental stage. Moreover, recent studies have shown how Fsh, but not 11-KT, is able to stimulate spermatogonial proliferation [38] adding strength to our findings. In particular, these authors evidenced how Fsh action is likely influenced by steroids but that these are not able to fulfill all Fsh functions necessarily required to initiate spermatogenesis.

Testicular expression of the estrogen receptor *erβ2* was significantly down-regulated (9-fold) in swimming seabass, suggesting again that swimming could result in the inhibition of mitotic spermatogonial division, also supported by the lower number of lsgA observed in this group. These results agree with the notion that estrogens, in particular E_2_ produced by the interstitial Leydig cells mainly under Fsh stimulation, have a pivotal role in regulating spermatogenesis in the early phases of spermatogonial proliferation through Sertoli cell intercession [55, 62]. In the teleost testis, estrogen receptors have a direct control on the expression of the Anti-Müllerian hormone (*amh*) [63] and high expression levels of *erβ1* and *erβ2* have been associated with early stages of precocious maturation in seabass males [64]. The observed lower mRNA expression levels of *amh* in swimmers vs. resters (5-fold), a factor known to induce spermatogonial stem-cell renewal and that is normally secreted together with *inhba* and *igf1* by Sertoli cells under Fsh-stimulated 11-KT production from Leydig cells [55], evidences a modulation of spermatogenesis that also affects the shift from mitotic to meiotic processes in the gonads of swimming fish.

In support of the hypothesis that not only the steroidogenic pathway but also factors involved in the regulation of spermatogonial proliferation may have been modulated by swimming, the expression of *smc1β*, a gene whose product is required for the maintenance of meiotic cohesion, was down-regulated in swimmers and presumably related to the detected decrease in *fshr* mRNA expression levels*.* Chauvignè et al. [65], found a steroid-dependent positive regulation of Smc1β production by gonadotropins at the same testis developmental stage (I-II) as the seabass in our study. Since this gene is expressed in germ cells surrounding the Sertoli cells and it has been found essential for the recombination between homologues during meiotic prophase [36], we propose that in swimmers the progress of meiosis may have been inhibited. This finding is also supported by the lack of primary spermatocytes found in the testes of the swimming group and importantly by the strong down-regulation (630-fold) of the expression of *gsdf1,* another gene involved in spermatogonial proliferation under Fsh-induced E_2_ release [55] and whose expression is restricted to Sertoli cells surrounding spermatogonia A [33, 65, 66]. Sawatari et al. [66] suggested a role for *gsdf1* in the proliferation of spermatogonial stem cells or undifferentiated spermatogonia A. Moreover, the observed down-regulation of its expression in testes from swimmers strengthens the idea that this gene may be a target candidate for puberty manipulation, although more accurate studies on its function and involved pathways have to be performed. Also, it has been found that both *gsdf* and *amh*, despite their demonstrated gonadotropic dependency [65], are under the control of progesterone [63] and we can speculate that their decreased expression in testis from swimmers may relate to the decreased expression levels of *3bhsd* observed in swimmers.

Finally, the expression of *inhba, a* member of the activin family that stimulates SgA proliferation since it encodes the active form of the dimeric protein activin A [67, 68], was down-regulated 20-fold in swimmers as compared to the non-exercised controls. Rolland and collaborators [69] suggested that in *O. mykiss,* the inhibition of *inhba* during more advanced spermatogenetic stages (III to V) would prevent the formation of a biologically active form of activin A, blocking germ cell proliferation and, consequently, permitting their meiotic differentiation. Conversely, our results suggest that in prepubertal seabass the down regulation of *inhba* expression by swimming is not by itself sufficient to trigger meiotic differentiation since *smc1β* expression was at the same time down-regulated and no primary spermatocytes were observed in swimmers. All the above-mentioned molecular data on testicular markers are in accordance with the histological observations in suggesting that swimming activity may have delayed spermatogenesis.

Overall, swimming activity may have operated by modulating the switch from an immature and non-receptive gonad to a hormonally competent one, thus affecting the BPG axis. One possible mechanism to try to explain the observed *fshr* down-regulation, and the subsequent pathways deriving from it leading to decreased testicular development, could be related to androgen-dependent dopaminergic regulation. Sex steroids have been shown to exert both negative and positive feedbacks on the gonadotropic/dopamine (DA) system through regulatory feedback [70]. The amplitude and the direction of this regulatory action is variable according to the development stage and the species analyzed [71–73]. As DA is also involved in the control of locomotion in fish [74, 75] and is able to inhibit the GnRH-induced pituitary gonadotropin production and, consequently, puberty [70], it may be hypothesized that this neurotransmitter may be at the base of the observed delay. Interestingly, previous studies have shown the tight relation between androgens and the dopaminergic system in another species [76] but to our knowledge no evidence is yet present for European seabass. It has to be mentioned that, currently, defining the role of DA in relation to early puberty and/or locomotor activity remains a challenging endeavour due to the complex physiological and adaptive roles that this signal can assume [54]. Another possible alternative or co-occurring mechanism underlying the observed reduction in testicular development by swimming could include a decline in the adiposity signal leptin due to reduced lipid levels caused by swimming as a high energy demanding activity. In European sea bass it has been shown that leptin has a positive effect on gonadotropin release [77]. However, there is no information on the possible regulation of leptin by swimming in teleosts. Obviously, further studies are required to clarify the roles of locomotor activity in determining the onset of puberty in teleosts.

## Conclusions

In conclusion, our results indicate that juvenile seabass males which were subjected to swimming exercise at optimal swimming speeds for 10 weeks experienced delayed testicular development. The role of several factors involved in testis development in this species was discussed with particular emphasis to their relation with swimming activity. Importantly, the results from this study strongly suggest that forced swimming exercise, during the onset of testicular development, can therefore be used as a natural and non-invasive treatment to inhibit testicular development. This study shows the promise for swimming-induced exercise in reducing the incidence of sexually precocious males with the resulting improvement in productivity and cost reduction in seabass aquaculture.

## Abbreviations

11-KT: 11-ketotestosterone
*11βhsd*: *11-beta hydroxysteroid dehydrogenase*
*3βhsd*: *3-beta-hydroxysteroid dehydrogenase*
ADV: Acoustic Doppler velocimeter
*amh*: *Anti-mullerian hormone*
BPG: Brain-pituitary-gonad
BW: Body weight
COT: Cost of transport
COT_min_: Minimal cost of transport
DA: Dopamine
*erβ2*: *Estrogen receptor-beta*
ESTs: Expressed sequence tags
*fshr*: *Follicle stimulating hormone receptor*
*gsdf1*: *Gonado-somal derived factor 1*
GSI: Gonadosomatic index
GW: Gonad weight
Hct: Hematocrit
*inhba*: *Inhibin beta A*
K: Fulton’s condition factor
lsgA: Late spermatogonia A
MBBR: Moving bed biofilm reactor
O_2_: Oxygen
RAS: Recirculating Aquaculture System
sc1: Primary spermatocytes
sgA: Spermatogonia
SL: Standard length
*smc1β*: *Structural maintenance of chromosomes protein 1B*
U_crit_: Critical swimming speed
U_opt_: Optimal swimming speed
